# A dual-radiomics model for overall survival prediction in early-stage NSCLC patient using pre-treatment CT images

**DOI:** 10.3389/fonc.2024.1419621

**Published:** 2024-08-14

**Authors:** Rihui Zhang, Haiming Zhu, Minbin Chen, Weiwei Sang, Ke Lu, Zhen Li, Chunhao Wang, Lei Zhang, Fang-Fang Yin, Zhenyu Yang

**Affiliations:** ^1^ Medical Physics Graduate Program, Duke Kunshan University, Kunshan, Jiangsu, China; ^2^ Department of Radiotherapy & Oncology, The First People’s Hospital of Kunshan, Kunshan, Jiangsu, China; ^3^ Deparment of Radiation Oncology, Duke University, Durham, NC, United States; ^4^ Radiation Oncology Department, Shanghai Sixth People’s Hospital, Shanghai, China

**Keywords:** early-stage non-small cell lung cancer, overall survival, explainable AI, radiomics, deep learning, radiation therapy

## Abstract

**Introduction:**

Radiation therapy (RT) is one of the primary treatment options for early-stage non-small cell lung cancer (ES-NSCLC). Therefore, accurately predicting the overall survival (OS) rate following radiotherapy is crucial for implementing personalized treatment strategies. This work aims to develop a dual-radiomics (DR) model to (1) predict 3-year OS in ES-NSCLC patients receiving RT using pre-treatment CT images, and (2) provide explanations between feature importanceand model prediction performance.

**Methods:**

The publicly available TCIA Lung1 dataset with 132 ES-NSCLC patients received RT were studied: 89/43 patients in the under/over 3-year OS group. For each patient, two types of radiomic features were examined: 56 handcrafted radiomic features (HRFs) extracted within gross tumor volume, and 512 image deep features (IDFs) extracted using a pre-trained U-Net encoder. They were combined as inputs to an explainable boosting machine (EBM) model for OS prediction. The EBM’s mean absolute scores for HRFs and IDFs were used as feature importance explanations. To evaluate identified feature importance, the DR model was compared with EBM using either (1) key or (2) non-key feature type only. Comparison studies with other models, including supporting vector machine (SVM) and random forest (RF), were also included. The performance was evaluated by the area under the receiver operating characteristic curve (AUCROC), accuracy, sensitivity, and specificity with a 100-fold Monte Carlo cross-validation.

**Results:**

The DR model showed highestperformance in predicting 3-year OS (AUCROC=0.81 ± 0.04), and EBM scores suggested that IDFs showed significantly greater importance (normalized mean score=0.0019) than HRFs (score=0.0008). The comparison studies showed that EBM with key feature type (IDFs-only demonstrated comparable AUCROC results (0.81 ± 0.04), while EBM with non-key feature type (HRFs-only) showed limited AUCROC (0.64 ± 0.10). The results suggested that feature importance score identified by EBM is highly correlated with OS prediction performance. Both SVM and RF models were unable to explain key feature type while showing limited overall AUCROC=0.66 ± 0.07 and 0.77 ± 0.06, respectively. Accuracy, sensitivity, and specificity showed a similar trend.

**Discussion:**

In conclusion, a DR model was successfully developed to predict ES-NSCLC OS based on pre-treatment CT images. The results suggested that the feature importance from DR model is highly correlated to the model prediction power.

## Introduction

Lung cancer is the leading cause of cancer death worldwide (1, 2). Non-small cell lung cancer (NSCLC) is the most common type, accounting for 80% of all lung cancers (3). Early-stage NSCLC (ES-NSCLC) refers to stages I and II of the disease (4), and over one-fifth of NSCLC cases were detected at ES-NSCLC (5). Surgery is the current standard-of-care treatment strategy for ES-NSCLC (3, 6, 7), while only approximately 70% of ES-NSCLC patients receive surgical treatment (8, 9). The remaining patients opt out of surgery due to medical or technical inoperability or personal preferences (10, 11). Radiation therapy (RT) has emerged as a standard noninvasive alternative to surgical resection (10). RT has been showing promising results in treating ES-NSCLC with high safety and efficiency (11). Nonetheless, the reported 3-year overall survival (OS) rate following RT varies widely, typically ranging from 39% ± 10% (12–14). Therefore, accurate OS prediction is thus crucial for personalizing medical treatment and informing decisions for optimizing therapeutic strategies.

Medical image-based OS prediction has been recently intensively studied and shown promising accuracy (15–17). Techniques such as radiomics and deep learning analyze image data to identify patterns that may be associated with underlying physiological and pathological conditions. Radiomic analysis, in particular, is a widely used non-invasive imaging quantification method (18). Typically, radiomics first determines the volume-of-interest (VOI), e.g., tumor in OS prediction tasks, and extracts the features that are defined based on experts’ domain knowledge to quantitatively capture the intensity, shape, size or volume, and texture information of VOIs, namely handcrafted radiomic features (HRFs) (17, 19). The extracted HRFs can be considered as the potential biomarkers that reflect patient underlying pathophysiology, and the classic machine learning classifiers can be employed to establish the correlations between OS and HRFs (20, 21). Several pilot studies have shown that the radiomic analysis based on the pre-treatment CT has the potential to accurately predict OS in ES-NSCLC following RT treatment (22, 23). Deep learning is a new approach for image quantification and characterization (24). Deep learning networks, which consist of multi-layer feed-forward neural networks, can be trained end-to-end in a supervised manner using medical images paired with observed OS data (19, 25). Through hierarchical progressive operations on the images, deep learning networks learn the high-level abstractions that capture the intrinsic representation of the image linking the input image to the outcome (26). These hidden high-level abstractions can also be explicitly derived from the trained deep learning models as the image deep features (IDFs) (26, 27). Deep learning methods have successfully demonstrated their effectiveness in OS prediction in ES-NSCLC following RT treatment (28, 29).

As HRFs are manually defined to capture specific characteristics within VOIs (17, 19), and IDFs are learned automatically from image data to discover complex patterns that may not be easily defined manually (19, 25), the combination of HRFs and IDFs has been recently investigated as a popular research direction (25, 30). Combining HRFs and IDFs can leverage the strengths of both approaches and has been shown to enhance the overall predictive accuracy and robustness of models (19, 31–33). As different image feature sources can be combined and fed into the model, the explanation and identification of key features are thus crucial for explaining the OS prediction mechanisms, optimizing feature extraction, and potentially improving model performance (34, 35). However, few studies have focused on evaluating the importance of each radiomic feature source and quantitatively explaining their contributions to the final prediction performance. The ML models producing state-of-the-art results possess a black-box nature; they use non-linear and nested fashion to process data, making them challenging to explain straightforwardly to humans (36). Although several explainability techniques have been proposed, including Local Interpretable Model-agnostic Explanations (LIME) (37), and Shapley Additive exPlanations (SHAP) (38), these explainability techniques each exhibit distinct limitations (39). For instance, LIME-generated explanations may be highly sensitive to minor variations in input data, potentially leading to inconsistent and unreliable explanations for the instance (39–41). SHAP has a high computational complexity, often requiring the use of various approximations of Shapley values, which may result in misleading explanations (38, 42). Recently, explainable boosting machine (EBM) has emerged as a promising alternative. EBM is designed to train on a single feature at a time in a round-robin fashion using a low learning rate (43, 44). As such, the effect of co-linearity between features can be mitigated, and the optimal feature function can be learned independently for each feature. Therefore, EBM maintains comparable prediction accuracy to state-of-the-art ML models while providing transparency in its decision-making process (43, 45).

This work developed a dual-radiomics (DR) model based on EBM to (1) predict 3-year OS in ES-NSCLC patients undergoing RT using pre-treatment CT images, and (2) provide explanations to the relationship between feature importance and prediction performance. In this work, 1) the handcrafted radiomic features (HRFs) extracted from gross tumor volume (GTV) and 2) image deep features (IDFs) extracted by a pre-trained Convolutional Neural Network (CNN) model were combined, and a novel EBM classifier was adopted for OS prediction as well as direct feature importance explanation.

## Materials and methods

### Imaging data

This work resorted to 132 RT patients with ES-NSCLC from the publicly available The Cancer Imaging Archive Lung1 (TCIA Lung1) dataset (46).

**Figure 1:**
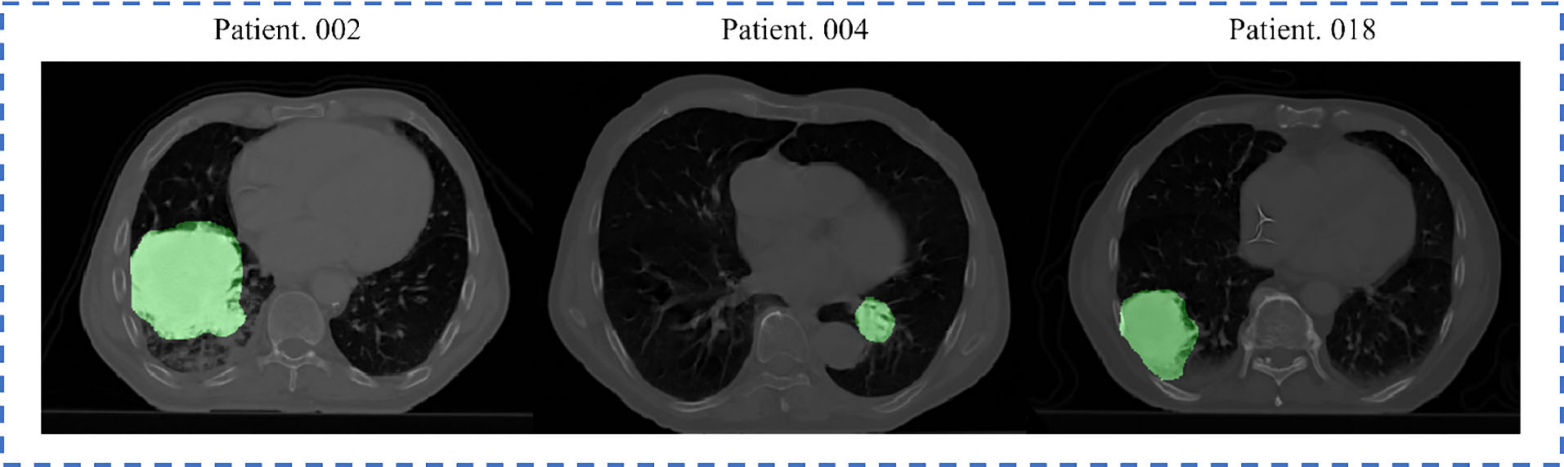
Representative slices from three Lung1 patients, with delineated GTV overlaid. The brighter areas on the figure represent the GTVs.

All patients underwent an FDG PET-CT scan for RT treatment planning. A spiral CT covering the entire thoracic region was acquired for each patient, with a resolution of 0.97 (mm) × 0.97 (mm) × 3 (mm, slice thickness). The GTV delineations for all patients were performed by experienced radiation oncologists on fused PET-CT images. The standard clinical delineation protocol with fixed window level settings of both CT (lung W1700; L-300, mediastinum W600; L40) and PET scan (W30000; L15000) was used for delineation (46). **Figure 1** displays a representative CT slice for several patients, each with the GTV delineations superimposed on it. All pre-treatment CT images and corresponding GTV masks were resampled to 1 × 1 × 1 mm^3^ isotropic voxel size for the following radiomic analysis and modelling. Based on the survival outcome (46), 89 and 43 patients were identified as under and over the 3-year OS group, respectively. All methods concerning the acquisition and usage of this dataset were in accordance with relevant guidelines and regulations.

### DR model design

**Figure 2:**
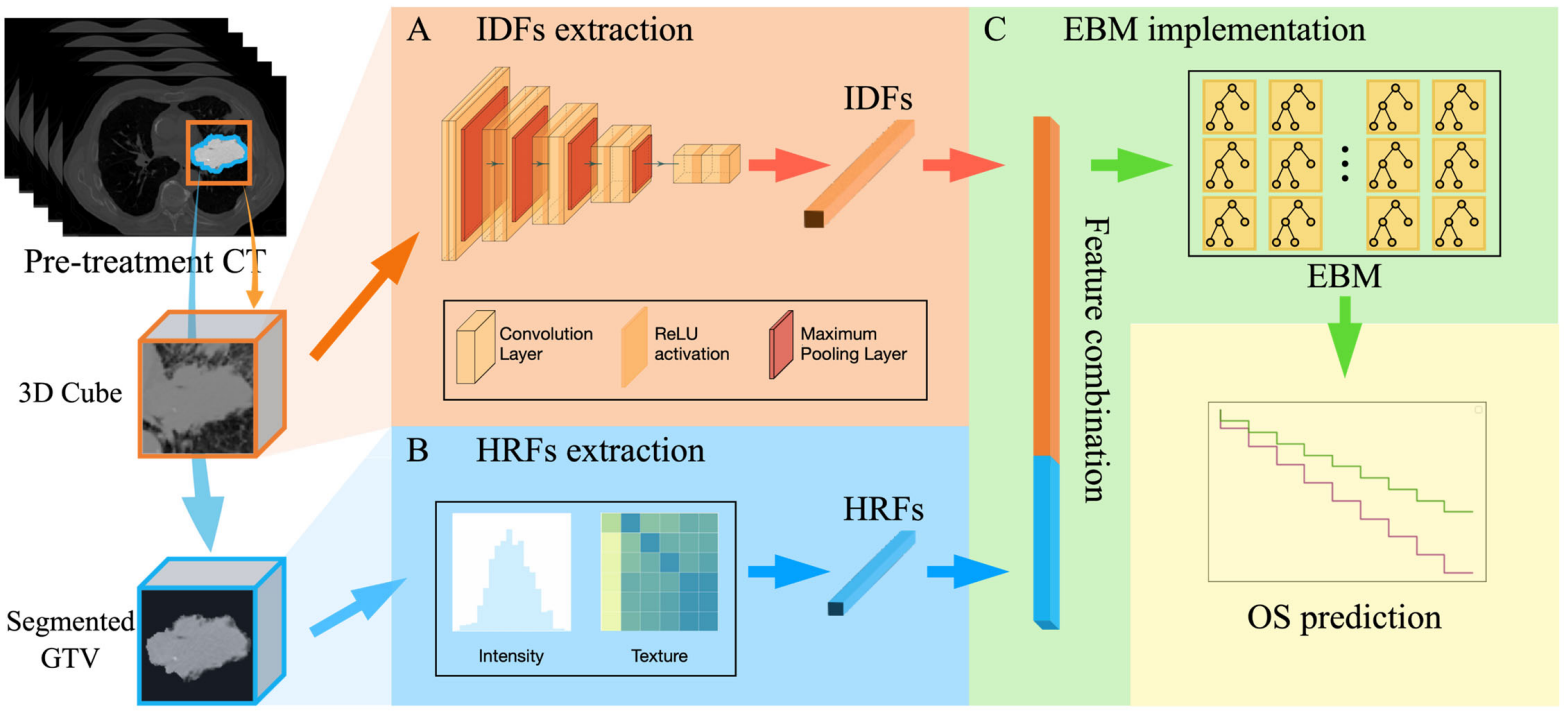
The overall design of the DR model, including three key steps: **(A)** IDFs extraction workflow using a pre-trained CNN-based encoder; **(B)** HRFs extraction that extracts 56 radiomic feature from segmented GTV; **(C)** EBM implementation for OS prediction (i.e., over 3-year OS or under).

The overall design of the proposed DR model is shown in **Figure 2**, which includes three key steps: (1) IDF extraction, (2) HRF extraction, and (3) EBM modelling for OS prediction.

#### IDFs extraction

The workflow for extracting image deep features (IDFs) is illustrated in **Figure 2A**. The input for this process was a GTV-centered 20×20×20 cm³ cube, which encompassed the GTV and surrounding tissue. Given the intricate and repetitive nature of patient anatomy of medical images, deep learning models are able to autonomously learn generic anatomical representations through self-supervision (30, 47). In this study, a publicly available pre-trained Models Genesis CNN U-Net encoder was employed (30). Models Genesis leverages self-supervised learning techniques to understand and extract anatomical features across multiple medical image datasets and has been reported to achieve state-of-the-art results in various medical image reorganization tasks (30, 48, 49). Specifically, the encoder includes four convolutional blocks, each comprising two convolutional layers followed by a max pooling layer. This architecture reduces the spatial dimension of the input image while increasing the feature information, resulting in a 512-channel deep image feature tensor representation. A global average pooling layer was then employed to average out each feature channel, forming the 512-dimensional feature vector. Therefore, by utilizing the pre-trained weights from Models Genesis to initialize the U-Net encoder, raw images were encoded into 512-dimensional feature vectors as IDFs. In addition, we utilized the CNN U-Net encoder that was trained from scratch to investigate the impact of different IDF extraction methods, i.e., pre-trained encoder vs. encoder trained from scratch. The entire deep feature extraction workflow was implemented in TensorFlow environment version 2.6.0.

#### HRFs extraction

**Figure 2B** illustrates the HRF extraction workflow. The 3D GTV volumes were first segmented from the pre-treatment CT images for all patients as the VOI. Compared to the 3D cube used in the IDFs extraction, the segmented GTV volume contains only the GTV itself following standard radiomic processing workflow (26, 50, 51). For each patient, 56 HRFs were extracted to capture the intensity and texture characteristics within the segmented GTV. These HRFs (**Table 1**) can be categorized into three groups according to their distinct joint density functions: 18 intensity-based features, 22 gray-level co-occurrence matrix (GLCM)-based features, and 16 gray-level run length matrix (GLRLM)-based features. Following the standard radiomic analysis pipeline (26, 50, 51), the intensity-based features were extracted using the raw GTV images; the second-order features (i.e., GLCM-based and GLRLM-based features) were derived from discretized images, which were obtained by employing a fixed bin number (=32) discretization to the resampled GTV images. The radiomic calculation was performed based on our in-house radiomics calculation platform using MATLAB (MATLAB R2022a; MathWorks, Natick, MA) (52). The entire feature extraction workflow has been fully calibrated against the image biomarker standardization initiative (IBSI) (53, 54).

**Table 1:** Fifty-six radiomic features included in this study.

**Intensity-based features**	1	Mean	**Gray level co-occurrence matrix (GLCM)-based features**	29	Inverse Difference
	2	Variance		30	Inverse Difference Moment
	3	Skewness		31	Info Measure Correlation 1
	4	Intensity histogram kurtosis		32	Info Measure Correlation 2
	5	Median		33	Inverse Difference Moment Normalized
	6	Minimum grey level		34	Inverse Difference Normalized
	7	10th percentile		35	Inverse Variance
	8	90th percentile		36	Joint maximum
	9	Maximum grey level		37	Sum Average
	10	Interquartile range		38	Sum Entropy
	11	Range		39	Sum Variance
	12	Mean absolute deviation		40	Joint Variance
	13	Robust mean absolute deviation	**Gray level run-length matrix (GLRLM)-based features**	41	Short Run Emphasis
	14	Median absolute deviation		42	Long Run Emphasis
	15	Coefficient of variation		43	Gray Level Non-Uniformity
	16	Quartile coefficient of dispersion		44	Gray Level Non-Uniformity Normalized
	17	Energy		45	Run Length Non-Uniformity
	18	Root mean square		46	Run Length Non-Uniformity Normalized
**Gray level co-occurrence matrix (GLCM)-based features**	19	Auto Correlation		47	Run Percentage
	20	Cluster Prominence		48	Low Gray Level Run Emphasis
	21	Cluster Shade		49	High Gray Level Run Emphasis
	22	Cluster Tendency		50	Short Run Low Gray Level Emphasis
	23	Contrast		51	Short Run High Gray Level Emphasis
	24	Correlation		52	Long Run Low Gray Level Emphasis
	25	Differential Entropy		53	Long Run High Gray Level Emphasis
	26	Dissimilarity		54	Gray Level Variance
	27	Joint Energy		55	Run Length Variance
	28	Joint Entropy		56	Run Entropy

#### OS prediction with EBM implementation

**Figure 2C** summarizes the EBM implementation for OS prediction. For each patient, a 512-dimensional IDF vector was directly concatenated with a 56-dimensional HRF vector, resulting in a single 568-dimensional feature vector. The obtained vector was utilized as the input x for an EBM. Specifically, EBM is an inherent explainable model based upon Generalized Additive Models (GAMs) (45), which can be mathematically formed as:


(1) $$E_{y}=\;\beta_{0}+\sum^{}f_{i}\left(x_{i}\right)$$

where $\beta_{0}$ represents the bias term.

In contrast to linear and multiple linear regression models, GAMs do not assume a linear relationship between predictor features x and the response variable y . Instead, the prediction of y involves learning an intercept $\beta_{0}$ along with functions that describe the non-linear relationship between the y and each predictor feature $x_{i}$ . The coefficients in a multiple linear regression model are replaced with learned functions $f_{i}$ that are not restricted to linear relationships. As suggested by Equation 1, the separated functions are learned for each predictor feature $x_{i}$ independently, allowing for the separate explanation of the importance of each predictor feature $x_{i}$ . Therefore, the mean value of the sum of $\left|f_{i}\left(x_{i}\right)\right|$ across all samples, referred to as the EBM’s mean absolute score, can directly quantify the importance of predictor feature $x_{i}$ . A link function was adopted to give the final binary OS prediction results. The EBM was implemented in Python environment with the InterpretML library (43).

The OS prediction performance was evaluated using the area under the receiver operating characteristic curve (AUC_ROC_), accuracy, sensitivity, and specificity with a 100-fold Monte Carlo cross-validation. In the 100-fold Monte Carlo cross-validation, the model underwent independent training with 100 different versions of randomly assigned training and test sets, adhering to an 80%-20% split (i.e., 34 over 3-year and 71 under 3-year samples for training; 9 over 3-year and 18 under 3-year samples for test). The EBM’s mean absolute scores were calculated as feature importance for both HRFs and IDFs feature sources, respectively. The key feature source was identified by the higher mean importance score. The student’s *t*-tests were also conducted to compare the importance scores of HRFs and IDFs derived from EBM. All calculations were performed on a computation workstation equipped with a 16-core Intel Core i7-13700KF CPU at 3.4 GHz, 16 GB of RAM, and an Nvidia GeForce RTX 4070 graphics card.

### Comparison study

To investigate the prediction performance and the feature importance explanation of the DR model, another two classic machine learning-based models were investigated:

1. Supporting vector machine (SVM) model: the prediction model based on SVM with linear kernel function using the combination of 512 IDFs and 56 HRFs. The linear kernel SVM distinguishes different categories by finding a maximal margin hyperplane in the input feature space (55). Such the linear modeling approach can be expressed using a common functional form, $y=\sum_{i=1}^{N}c_{i}x_{i}+b$ (56), where $c_{i}$ is the coefficient and *b* is an offset constant. The coefficients $c_{i}$ were used as estimators of feature importance to identify the key feature source.
2. Random forest (RF) model: the prediction model based on RF using the combination of 512 IDFs and 56 HRFs. The key feature source in the RF model was estimated using Mean Decrease Impurity (MDI) (57, 58). The MDI calculates the contribution of each feature to the homogeneity of the nodes and leaves by averaging the decrease in impurity caused by splits on that feature across all trees in the forest, thereby providing a rough estimation of the feature’s overall importance.

To assess the effectiveness of feature importance explanations, the following comparison experiments were subsequently performed:

1. For the DR model, the model performance was compared with (1) EBM model with key feature source (identified by mean EBM score) as input, and (2) EBM with the rest feature source as input.
2. For the SVM model using both IDFs and HRFs, the performance was compared with (1) SVM model with key feature source (identified by mean SVM coefficient) as input, and (2) SVM with the rest feature source as input.
3. For the RF model using both IDFs and HRFs, the model performance was compared with (1) RF model with key feature source (identified by mean RF MDI) as input, and (2) RF with the rest feature source as input.

For all the above comparison experiments, AUC_ROC_, accuracy, sensitivity, and specificity evaluation matrices with 100-fold Monte Carlo cross-validation were also employed. The student’s t-tests were employed to measure the performance differences with a significance level of 0.01 when applicable.

## Results

**Figure 3:**
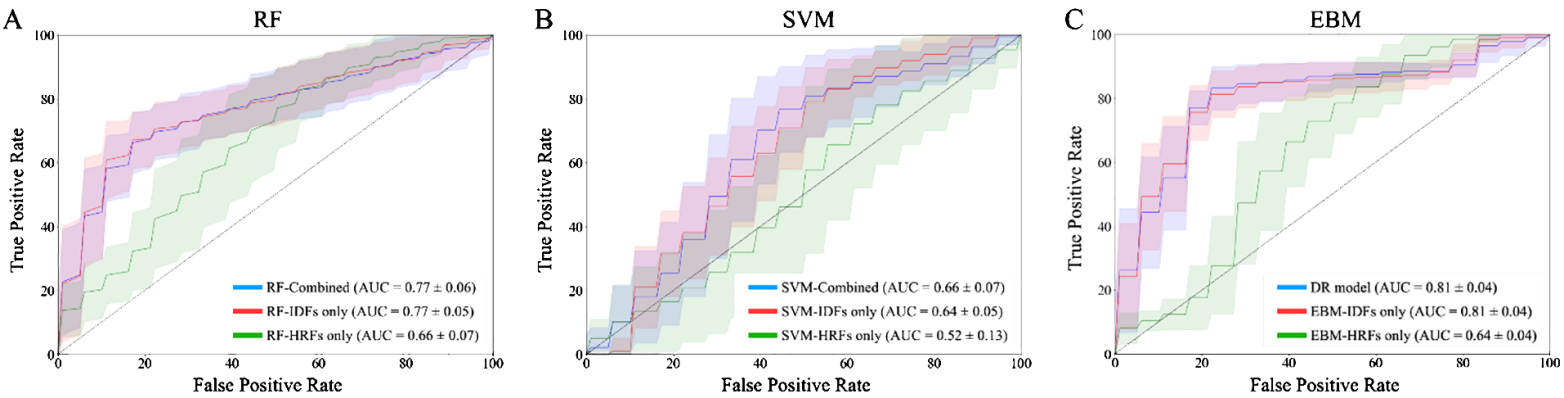
ROC curves of 100-fold Monte Carlo cross-validation from **(A)** RF-based models; **(B)** SVM-based models; **(C)** EBM-based models and our DR model. Green lines represent the models with HRFs only. Red lines represent models with IDFs only. Blue lines represent models with combined IDFs and HRFs.

**Table 2** provides the evaluation metrics results of comparative studies. The DR model demonstrated superior performance in predicting 3-year OS, with a mean AUC_ROC_ = 0.81, mean sensitivity = 0.75, mean specificity = 0.82, and mean accuracy = 0.80. EBM with IDFs only showed a comparable AUC_ROC_ and specificity to the DR model, and its sensitivity and accuracy were reduced at 0.73 and 0.79, respectively. EBM with HRFs only showed limited results, with a mean AUC_ROC_ = 0.64, mean sensitivity = 0.65, mean specificity = 0.61, and mean accuracy = 0.62. Both RF-based models and SVM-based models demonstrated the same trends, i.e., models with combined IDFs and HRFs outperforming models with IDFs or HRFs only. The RF model incorporating both HRFs and IDFs, alongside the RF with IDFs only, achieved the second-highest mean AUC_ROC_ (=0.77), while they exhibited limited sensitivity, measured at 0.30 and 0.29, respectively. All SVM-based models showed limited performances. **Figure 3** compares the ROC curves derived from (A) RF-based models, (B) SVM-based models, (C) EBM-based models, and our DR model. In **Figure 3**, green lines represent the models with HRFs only, red lines represent models with IDFs only, and blue lines represent models with combined features. **Table 3** shows the P-value results of the AUC_ROC_ comparison results, where marker “*” indicates the statistical significance (i.e., P-value<0.01).

**Table 2:** AUC_ROC_, sensitivity, specificity, and accuracy results of comparative studies.

	RF-based			SVM-based			EBM-based		
	HRFs only	IDFs only	Combined	HRFs only	IDFs only	Combined	HRFs only	IDFs only	DR model
**AUC_ROC_**	0.66 ± 0.07	0.77 ± 0.05	0.77 ± 0.06	0.52 ± 0.13	0.64 ± 0.05	0.66 ± 0.07	0.64 ± 0.04	**0.81 ± 0.04**	**0.81 ± 0.04**
**Sensitivity**	0.21 ± 0.06	0.29 ± 0.15	0.30 ± 0.13	0.36 ± 0.09	0.34 ± 0.15	0.25 ± 0.09	0.65 ± 0.21	0.73 ± 0.12	0.75 ± 0.10
**Specificity**	0.89 ± 0.06	0.96 ± 0.04	0.96 ± 0.04	0.72 ± 0.08	0.77 ± 0.07	0.81 ± 0.06	0.61 ± 0.09	0.82 ± 0.04	0.82 ± 0.04
**Accuracy**	0.67 ± 0.04	0.74 ± 0.05	0.75 ± 0.05	0.60 ± 0.06	0.64 ± 0.06	0.62 ± 0.05	0.62 ± 0.06	0.79 ± 0.04	0.80 ± 0.03

Bold values represent the best statistically significant AUC_ROC_ results.

**Table 3:** P-values of AUC_ROC_ results of the DR model compared to the RF-based models, SVM-based models, and EBM-based models in Monte Carlo cross-validation.

Model 1	DR							
Model 2	RF-HRFs only	RF-IDFs only	RF-combined	SVM-HRFs only	SVM-IDFs only	SVM-combined	EBM-HRFs only	EBM-IDFs only
**P-value**	0.00^*^	0.00^*^	0.00^*^	0.00^*^	0.00^*^	0.00^*^	0.00^*^	0.49

“*” marks the statistically significant difference (i.e., P-value < 0.01).

EBM scores suggested that IDFs held significantly greater importance (normalized average score = 0.0019) than HRFs (0.0008). This discrepancy in feature importance was reflected in the comparative analyses: the EBM model utilizing solely IDFs achieved a comparable AUC_ROC_ to the DR model, whereas the EBM with HRFs only exhibited a markedly reduced AUC_ROC_. In contrast, models based on RF and SVM achieved limited performance compared to the DR model. The importance scores derived from RF and SVM suggested that IDFs (0.0017/0.0016 in RF/SVM) showed lower importance than HRFs (0.0027/0.0031), which is not aligned with the model performance. This inversion contradicts the observed model performance trends. **Table 4** shows the P-value results for comparing importance scores of IDFs and HRFs derived from RF, SVM, and EBM, where marker “*” indicated the statistical significance (i.e., P-value<0.01).

**Table 4:** P-values for comparing importance scores of IDFs and HRFs derived from RF, SVM, and EBM.

**Group I**	**Importance scores of IDFs from RF**	**Importance scores of IDFs from SVM**	**Importance scores of IDFs from EBM**
**Group II**	**Importance scores of HRFs from RF**	**Importance scores of HRFs from SVM**	**Importance scores of HRFs from EBM**
**P-value**	0.00^*^	0.00^*^	0.00^*^

“*” marks the statistically significant difference (i.e., P-value < 0.01).

**Supplementary Table S1** in the **Supplementary Materials** summarized the prediction results based on the IDFs training from scratch. The EBM model with the combination of IDFs (trained from scratch) and HRFs achieved a mean AUC_ROC_ = 0.75, a mean sensitivity = 0.77, a mean specificity = 0.79, and a mean accuracy = 0.79. The EBM using only IDFs (trained from scratch) obtained a mean AUC_ROC_ = of 0.77, a mean sensitivity = 0.80, a mean specificity = 0.80, and a mean accuracy = 0.80. Both these two models were inferior to our DR model, and EBM with IDFs (from Model Genesis) also outperformed the EBM with IDFs (trained from scratch). **Supplementary Table S2** in the **Supplementary Materials** provides the 95% confidence interval of AUC_ROC_ for all comparative models.

## Discussion

In this study, we successfully developed a DR model to predict the OS of ES-NSCLC patients following RT based on pre-treatment CT images. Compared to the previous image-based ES-NSCLC OS prediction tasks (59, 60), our DR model achieved high prediction performance with a mean AUC_ROC_ of 0.81, sensitivity of 0.75, specificity of 0.82, and accuracy of 0.80. It also outperformed all comparative models (i.e., SVM-based models, RF-based models, and EBM-based models) in this study. The proposed DR model integrates both HRFs and IDFs, where (1) HRFs are manually created at a lower level of abstraction based on expert knowledge and predefined rules to capture specific and interpretable characteristics of the GTV (17, 19), and (2) IDFs automatically learn high-level abstractions through multiple layers of processing to capture intricate patterns in the GTV (25, 30). The prediction results suggested that the combination of features from different sources may leverage the strengths of both approaches and provide a more comprehensive description of image characteristics. It is worth mentioning that there are few existing studies that investigated ES-NSCLC patients from the TCIA Lung1 dataset. Consequently, there are no existing results that can be directly compared with our work. Although higher AUC_ROC_ values can be observed in other NSCLC OS prediction studies (24, 32, 33, 59–61), the comparisons between the state-of-the-art results for NSCLC OS prediction and our DR model should be interpreted with caution due to differences in cancer stages and datasets.

In this study, direct feature concatenation was employed to combine the HRFs and IDFs. Unlike more complex fusion methods such as feature transformation (62) or neural network-based fusion (63), direct concatenation preserves the transparency of the feature space. As EBM quantitatively evaluates each feature’s contribution using mean absolute score, each component of the concatenated vector thus directly corresponds to a specific feature source. Additionally, since decision trees partition the feature space based on simple thresholding rules (64), direct concatenation can be computationally efficient. The reliability of the feature importance has been proven by comparison studies. The DR model identified IDF as the key feature source, attributing to it a higher normalized average importance score (=0.0019) compared to HRF (scored 0.0008), and the P-value for the importance scores of IDFs and HRFs derived from EBM is 0.00. This discrepancy in feature importance is consistent with the model prediction performance, i.e., EBM with IDFs only achieved a comparable average AUC_ROC_ (=0.81) to the DR model, while EBM with HRFs only achieved a limited average AUC_ROC_ (=0.64). In contrast, RF and SVM models suggested an inverse feature importance, with IDFs registering lower importance scores (0.0017/0.0016 for RF/SVM, respectively) compared to HRFs (0.0027/0.0031) and the P-values for the importance scores of IDFs and HRFs derived from RF and SVM are both 0.00. Such results contradict the observed model performance trends, i.e., RF/SVM with IDFs only outperformed the RF/SVM with HRFs only. For the SVM model with linear kernel, feature coefficients were utilized to explain feature importance. The coefficients of individual features still might provide a misleading representation of their actual importance due to the relevance and interaction among features (36). For the RF model, MDI was used to assess feature importance. RF splits nodes based on decreases in feature impurity (65). If noise features coincidentally reduce impurity in specific data subsets, they can receive undeservedly high MDI scores (36, 57). This can introduce a systematic bias in the explanation of feature importance, potentially misleading the assessment (57). The feature importance explanation results suggested that the DR model based on EBM has demonstrated its effectiveness in explainability and can be potentially generalized in other multi-feature fusion studies for enhancing explanatory power.

In this work, the public available Model Genesis model was adopted as the pre-trained weights of the CNN U-Net encoder (30). The Model Genesis was trained using a large and diverse LUNA 2016 dataset (66) to learn broader and more generalized feature representations (25, 30). The pre-trained weight has been reported to show the state-of-the art performance on various medical image analysis tasks, including reducing false positives in detecting lung nodules (NCC) and pulmonary embolism (ECC) (30). To investigate the impact of the pre-trained encoder, the CNN U-Net encoder trained from scratch was also explored. The results suggested that EBM using IDFs that were trained from scratch showed limited performance, and the performance of EBM using the combination of IDFs (trained from scratch) and HRFs was also inferior to our DR model. Due to the limited sample size of our dataset, the model trained from scratch may have failed to learn strongly discriminative features effectively. Future investigation on other CNN or transformer-based pre-trained model are also needed to test the impact of deep learning image encoders. This study selected a subgroup of 132 patients with ES-NSCLC from the TCIA Lung1 dataset, rather than including all patients. The OS of patients with advanced or locally advanced NSCLC involves multiple factors, such as patient conditions (67), complications (e.g., second tobacco-caused neoplasms (67), chemotherapy-induced bone marrow failure (68)), treatment modalities (69), etc. The current TCIA Lung1 dataset lacks details on treatment procedures (e.g., lack of chemotherapy information). Therefore, OS modeling for advanced or locally advanced NSCLC can be challenging (70–73). Our analysis was finally restricted to the 132 patients with ES-NSCLC and primarily focused on technical development. Meanwhile, the choice of chemotherapy has been reported to depend on the patient’s genetics, cancer stage, and gender (74–77), and its administration could potentially impact patient OS outcomes (78–80). The lack of detailed chemotherapy information in the TCIA Lung1 dataset (i.e., unspecified details about which patients received chemotherapy) limits our ability to fully explain its impact on survival rates. Further research with comprehensive information (including chemotherapy, immunotherapy, etc.) is needed to test our model on patients with ES-NSCLC, advanced, or locally advanced NSCLC. Furthermore, the data distribution was 33% of patients with OS over 3 years and 67% with OS under 3 years; no severe data imbalance was observed in this task. Therefore, we did not include class-balancing approaches during the training process. In future work, class-balancing approaches can be explored to determine if they can further enhance model performance.

## Conclusion

In this work, we successfully developed a DR model to predict ES-NSCLC OS based on pre-treatment CT images, and the results suggested that feature importance from DR model is highly correlated to model prediction power. The proposed methodology can be generalized to other employing multi-feature fusion models to evaluate feature importance.

## Data Availability

The original contributions presented in the study are included in the article/**Supplementary Material**. Further inquiries can be directed to the corresponding author. All imaging data used in this study is obtained from the publicly available TCIA Lung1 dataset, which can be downloaded via request from the TCIA Lung1 website: https://wiki.cancerimagingarchive.net/display/Public/NSCLC-Radiomics.
