# Supplementary material for: A dual-radiomics model for overall survival prediction in early-stage NSCLC patient using pre-treatment CT images
Source: Front Oncol. 2024 Aug 14;14:1419621. doi: 10.3389/fonc.2024.1419621 (PMC11349529; doi:10.3389/fonc.2024.1419621)
Supplement: Supplementary file 1 [file Table1.docx]

Supplementary Materials

Supplementary Tables

Table SI. AUC_ROC_, sensitivity, specificity, and accuracy results of models using IDFs (scratch).

|  | **AUC_ROC_** | **Sensitivity** | **Specificity** | **Accuracy** |
| --- | --- | --- | --- | --- |
| **EBM with IDFs (scratch) only** | 0.77 ± 0.02 | 0.80 ± 0.03 | 0.80 ± 0.09 | 0.80 ± 0.03 |
| **EBM-combined IDFs (scratch) and HRFs** | 0.75 ± 0.02 | 0.79 ± 0.03 | 0.77 ± 0.11 | 0.79 ± 0.03 |

Table SII. 95% confidence interval of AUC_ROC_ values for all comparative models.

| **Model** | **95% confidence interval of AUC_ROC_** |
| --- | --- |
| RF-HRFs only | 0.65 – 0.67 |
| RF-IDFs only | 0.76 – 0.78 |
| RF-combined IDFs and HRFs | 0.76 – 0.78 |
| SVM-HRFs only | 0.49 – 0.55 |
| SVM-IDFs only | 0.63 – 0.65 |
| SVM-combined IDFs and HRFs | 0.65 – 0.67 |
| EBM-HRFs only | 0.63 – 0.65 |
| EBM-IDFs only | 0.80 – 0.82 |
| DR model | 0.80 – 0.82 |
| EBM-SDFs only | 0.77 – 0.77 |
| EBM-combined SDFs and HRFs | 0.75 – 0.75 |
